# Chickpea Ferritin CaFer1 Participates in Oxidative Stress Response, and Promotes Growth and Development

**DOI:** 10.1038/srep31218

**Published:** 2016-08-09

**Authors:** Shaista Parveen, Deepti Bhushan Gupta, Suchismita Dass, Amit Kumar, Aarti Pandey, Subhra Chakraborty, Niranjan Chakraborty

**Affiliations:** 1 National Institute of Plant Genome Research, Aruna Asaf Ali Marg, New Delhi-110067, India; 2 Department of Biotechnology, TERI University, New Delhi, India

## Abstract

Ferritins store and sequester iron, and regulate iron homeostasis. The cDNA for a stress-responsive phytoferritin, previously identified in the extracellular matrix (ECM) of chickpea (*Cicer arietinum*), was cloned and designated *CaFer1*. The *CaFer1* transcript was strongly induced in chickpea exposed to dehydration, hypersalinity and ABA treatment. Additionally, it has role in the defense against *Fusarium oxysporum* infection. Functional complementation of the yeast frataxin-deficient mutant, *Δyfh1*, indicates that CaFer1 functions in oxidative stress. The presence of CaFer1 in the extracellular space besides chloroplast establishes its inimitable nature from that of other phytoferritins. Furthermore, CaFer1 expression in response to iron suggests its differential mechanism of accumulation at two different iron conditions. CaFer1-overexpressing transgenic plants conferred improved growth and development, accompanied by altered expression of iron-responsive genes. Together, these results suggest that the phytoferritin, CaFer1, might play a key role in maintenance of iron buffering and adaptation to environmental challenges.

Iron acts as a redox component and forms an integral part of vital biological processes that include electron transfer, oxygen transport, oxidative phosphorylation, and DNA biosynthesis. However, excess iron increases the level of reactive oxygen species (ROS)^1^ by participating in the generation of hydroxyl radicals via Fenton reaction^2^. Thus, plants strictly control iron metabolism to avoid iron-deficiency or iron-overload. Moreover, the regulation of iron homeostasis influences oxidative stress tolerance^3^ and may affect the responses to other abiotic stresses. The cellular antioxidant defense system and the tight regulation of iron homeostasis can reduce production of oxyradicals.

Regulation of iron homeostasis includes strict control of iron assimilation. In non-graminaceous plants, a low-iron-inducible plasma membrane bound ferric-chelate reductase mobilizes iron from the soil, and ZIP family IRT transporters transport Fe^2+^ into root cells. In grasses, iron-chelating phytosiderophores release into the rhizosphere and form Fe^3+^-MA (mugineic acid) complexes, and are taken up by specific membrane-bound high-affinity transporters^4^. Tight regulation of iron homeostasis also depends on ferritins, the multi-subunit proteins that store and sequester iron^5^. Ferritin can store up to 4500 Fe (III) atoms in its central cavity^6^ and can release the stored Fe when the need arises^7^. Ubiquitously present in bacteria, plants and animals^8^,^9^,^10^, ferritins have highly conserved sequences and structure^11^. Their high sequence conservation among eukaryotes implies that they might share a common ancestral origin^11^,^12^. Hyde *et al.*^13^ reported the first phytoferritin gene in peas and since then, ferritins have been identified and cloned in many plants^14^.

Emerging research indicates the important role of phytoferritins in iron homeostasis. For example, *Arabidopsis* ferritin T-DNA mutants show impaired growth and development in response to iron stress^15^. Animals mainly regulate ferritin synthesis at the translational level, but plants regulate ferritin synthesis at the transcriptional level^12^,^16^,^17^. Environmental cues, such as pathogen attack^18^, photoinhibition^19^, ABA^20^, and oxidative stress^3^ affect phytoferritin transcription. Yet, it remains unclear whether the products of the multiple ferritin genes in plants have divergent or conserved functions.

Secreted proteins, circulating in the extracellular space, are readily accessible to external stimuli and thus are extremely attractive targets for stress adaptation. Extracellular proteins play important roles in Fe acquisition, host-pathogen interactions and defense against pathogen infection. In Chlamydomonas, the secreted proteins FEA1 and FEA2 bind iron and facilitate Fe uptake^21^. In insects, secreted ferritin functions as an iron transporter^22^. Animal cells secrete ferritins into the cerebrospinal and synovial fluid^23^, but no extracellular ferritin has yet been characterized in plants.

Dedicated protein components present in different cell organelles function to protect plants from adverse environmental conditions, and protein functions are intimately linked with their compartmentalization. Thus, investigation of the stress-responsive proteome profile and protein localization can help us understand the mechanisms of stress adaptation. For example, phytoferritins localize to their site of action and influence regulated gene expression in different developmental stages and environmental conditions^15^. Our earlier study on the global analysis of the ECM of chickpea revealed the presence of ferritin^24^, which had never been reported to be associated with this compartment. This was further strengthened by the observation of ferritins, differentially regulated under dehydration, in the ECM of two contrasting cultivars of chickpea differing in degree of tolerance^25^,^26^. In this study, we screened the chickpea cDNA library and cloned the stress-responsive ferritin, designated *CaFer1*. Our results suggest that CaFer1 might play key roles in iron homeostasis, stress tolerance and plant growth and development.

## Results

### Molecular cloning and structural organization of *CaFer1*

**Figure 1:**
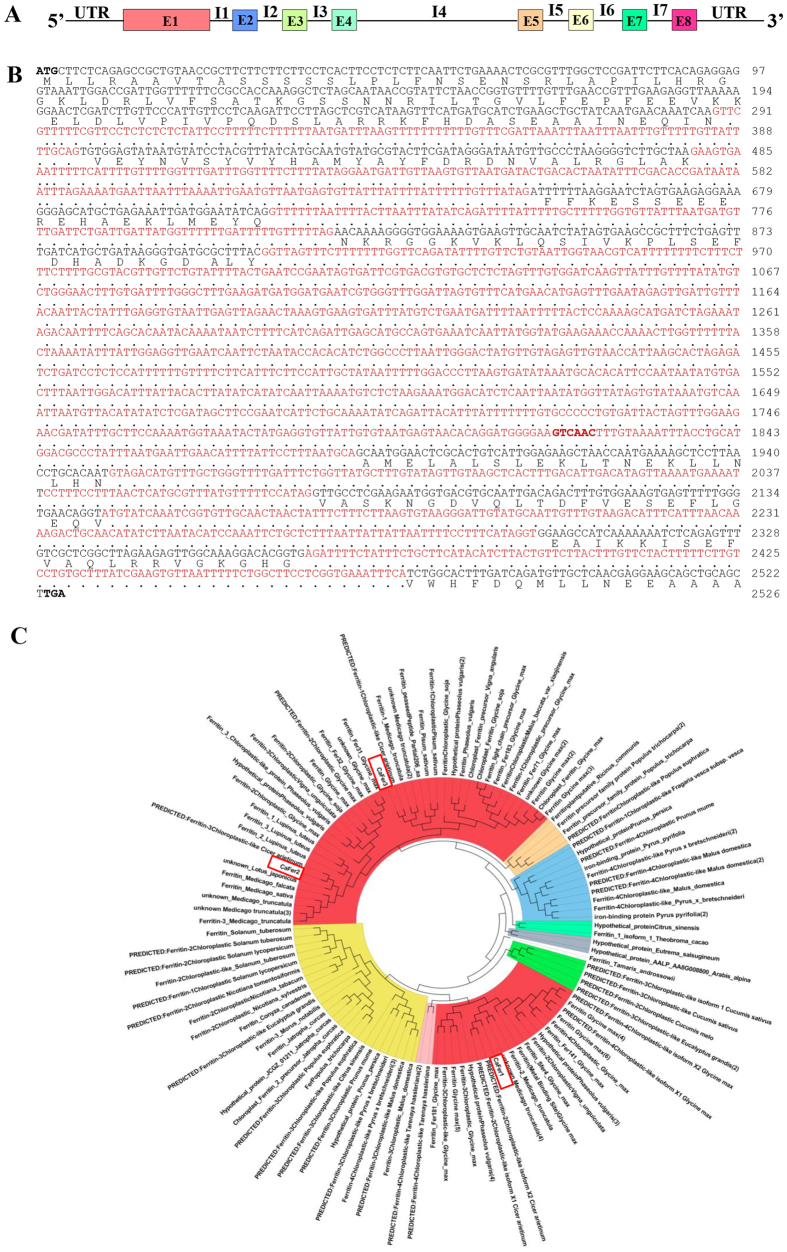
Structural organization of *CaFer1* and phylogenetic analysis. (**A**) Schematic representation of the genomic organization of *CaFer1*. E, exon; I, intron; UTR, untranslated region. (**B**) Nucleotide sequence of *CaFer1* containing introns (red) and the open reading frame. The positions of initiation codon (ATG) and stop codon (TGA) are indicated in bold letters. (**C**) Phylogenetic tree, constructed by neighbour-joining method, showing the relationship of chickpea ferritins (boxed) with other phytoferritins. Phylogenetic relationships of ferritins from different kingdoms are shown by different colours for each clade. Ferritins from bacteria, fungi, insects, plants and animals are denoted by blue, pink, light green, green and orange, respectively.

In our previous study, we analyzed the dehydration response of ECM of chickpea^25^ and quantified the temporal changes in the proteome profile. The proteomic analysis led to the identification of ferritin/s on the basis of homology-based search from *Vigna unguiculata*. The protein spots corresponding to ferritin/s displayed differential accumulation pattern under dehydration (Supplementary Fig. 1). This indicated, for the first time ever, the presence of phytoferritin in the extracellular space, which might have key role/s in iron metabolism and stress adaptation. Consequently, it prompted us to characterize the novel ECM-specific ferritin. Towards this end, we designed 5′ degenerate primers from the conserved region that included the sequence of the tag obtained from MS/MS analysis and performed 3′ RACE PCR. The PCR analysis yielded a 0.6 kb amplicon. Next, we screened a normalized cDNA library using the 0.6 kb fragment as probe and isolated a full-length cDNA, henceforth referred to as *CaFer1* (*Cicer arietinum* ferritin 1)^27^. Sequence analysis revealed that the *CaFer1* transcript is 995 bp, with 765 bp coding region, 88 bp 5′-UTR, and 142 bp 3′-UTR. The cDNA encodes a 254 amino acid protein with an approximate molecular weight of 28 kDa. To further characterize *CaFer1*, we assigned intron positions by comparing the corresponding genomic fragment of 2.526 kb with the cDNA sequence (Fig. 1A,B). The intron junctions showed the consensus sequence of dN/GT… AG/N and shared the same positions as reported for maize ferritin^28^. However, the sequences of the introns are not conserved and the introns in *CaFer1* vary from 106–982 bp (Fig. 1B).

### Evolutionary relationship of chickpea ferritins with other phytoferritins

We screened the Chickpea Transcriptome Database [CTDB (http://www.nipgr.res.in/ctdb.html)], which led to the identification of two more forms of ferritin, referred to as CaFer2 (NCBI accession number: gi|502147479:108–890) and CaFer3 (NCBI accession number: gi|502104289:1–777). Multiple sequence alignment revealed 62% homology among these forms. CaFer2 and CaFer3 both are 28 kDa proteins, having 783 and 777 nucleotides, respectively. BLAST analysis with the chickpea draft sequence^29^ showed *CaFer1* at a single locus on chromosome 2, and *CaFer2* and *CaFer3* on chromosome 6 and chromosome 3, respectively. Further, we checked the phylogenetic relationship of the three chickpea ferritins with other phytoferritins. A phylogram was constructed by selecting the top 50 hits of NCBI BLAST search program, which displayed eight different clades (Fig. 1C). Legume species were arranged in two different clades. Ferritin of *Lotus japonicus* exhibited common ancestry with CaFer2, but CaFer1 and CaFer3 both shared their homology with ferritin from *Medicago truncatula*. CaFer2 and CaFer3 were placed in the same clade, while CaFer1 was placed in a separate clade. The phylogenetic divergence may be attributed to the different function of CaFer1 from that of CaFer2 and CaFer3, while all three showed evolutionary relation with legume ferritins.

### Tissue-specific and abiotic stress-responsive expression of *CaFer1*

Tissue-specific expression provides crucial functional information for stress-responsive genes. We examined the tissue-specific expression of *CaFer1* by Northern blot analysis using RNA from root, stem, leaf, flower and pod of 3-week-old chickpea seedlings. The transcript abundance showed the highest expression in pods, and moderate expression in root and stem as compared to leaf (Supplementary Fig. 2A).

**Figure 2:**
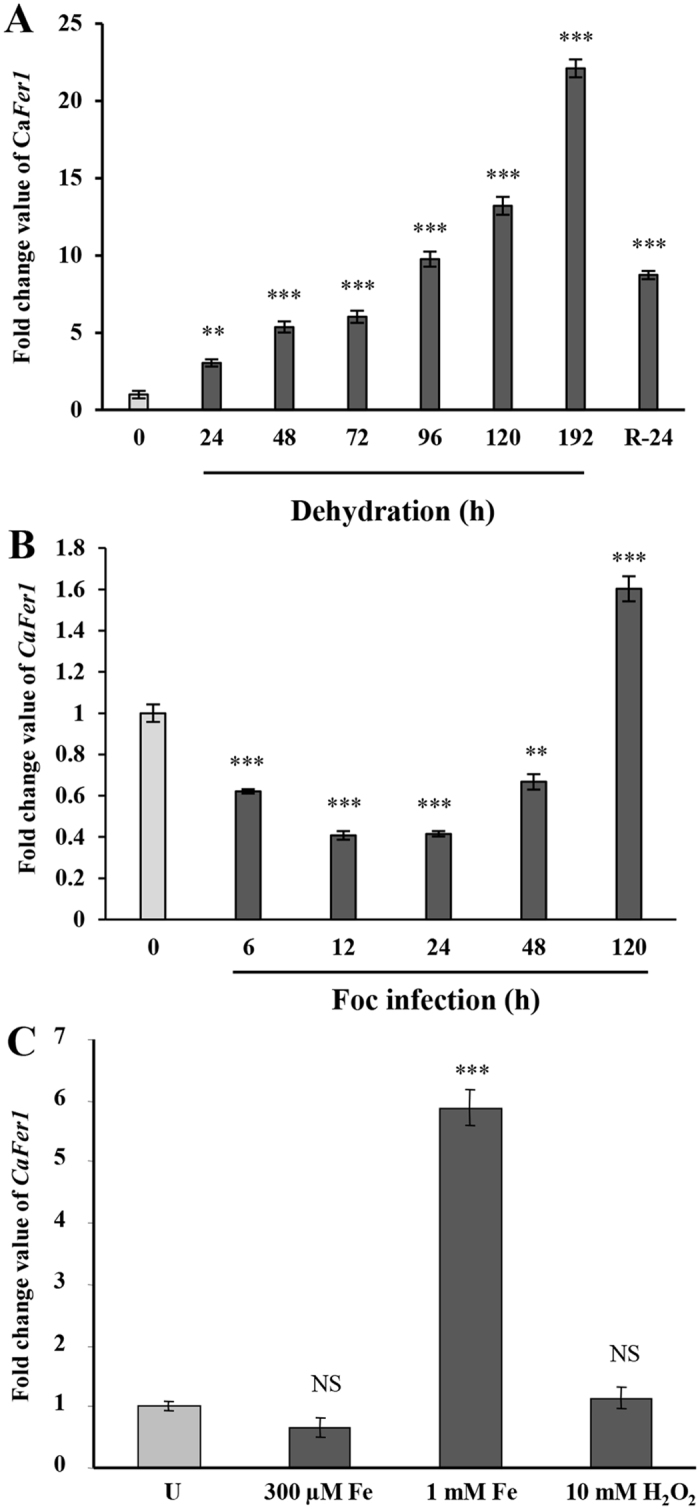
Stress-responsive altered expression of *CaFer1*. (**A**) Time-dependent accumulation of *CaFer1* transcript in response to dehydration, as indicated. (**B**) *Fusarium oxysporum f. sp. ciceris* (Foc) effected altered expression of *CaFer1*. (**C**) qRT-PCR analysis showing differential accumulation of *CaFer1* transcript in response to Fe (300 μM and 1 mM) and 10 mM H_2_O_2_. The expression levels of *CaFer1* were normalized to expression of EF1α. Error bars represent the SE from three replicates. Asterisks indicate significant differences relative to the untreated background (Student’s t test). NS, not significant; U, untreated.

As water-deficit responsive function of phytoferritin is known^28^; we sought to know the dehydration-responsive behavior of *CaFer1*. The transcripts were progressively induced in response to dehydration with maximal induction at 192 h. It is likely that the *CaFer1* transcripts induced slowly due to severe damage inflicted to the seedlings. However, in later time points, the transcripts involved in stress adaptation might have maximally induced to help overcome the damage (Fig. 2A).

The dehydration-responsive pathway often overlaps with responses to hypersalinity and ABA and therefore, we examined *CaFer1* expression in response to these conditions. The transcript abundance increased with higher concentrations (250–500 mM) of NaCl (Supplementary Fig. 2B). Treatment of seedlings with 100 μM ABA led to strong expression of *CaFer1* after 2 h (Supplementary Fig. 2C). These results suggest that *CaFer1* may function in multiple stress responses, possibly through ABA-dependent pathway.

### Involvement of *CaFer1* in pathogen and oxidative stress response

Ferritins have earlier been implicated in biotic stress responses^18^,^30^. To test if *CaFer1* has a role in pathostress response, we examined *CaFer1* transcript in chickpea seedlings challenged with *Fusarium oxysporum* f. sp. *ciceris* (Foc). *CaFer1* expression was suppressed in the early phase, i.e., 6–24 h post-infection (Fig. 2B), corresponding to a possible period of activation of programmed cell death (PCD) and decreased ROS scavenging capacities. However, transcript abundance increased thereafter, until 120 h of infection, possibly to avoid ROS-induced cellular damage.

To determine the iron-responsive function of *CaFer1* and its role in oxidative stress, we quantified the expression using different concentrations of Fe (300 μM for moderate iron supplement and 1 mM for iron overload) and 10 mM H_2_O_2_. The *CaFer1* transcript abundance remained unchanged at 300 μM Fe, but increased at 1 mM Fe (Fig. 2C). However, treatment with 10 mM H_2_O_2_ did not lead to any change in *CaFer1* expression (Fig. 2C). These results indicate that *CaFer1* has oxidative stress-responsive function under iron overload condition. The unchanged expression of *CaFer1* at 300 μM Fe is intriguing, which indicates its differential mechanism of accumulation at varying iron concentrations. The unaltered level of *CaFer1* at 10 mM H_2_O_2_ showed that *CaFer1* is not affected by H_2_O_2_ at transcriptional level.

### *CaFer1* can rescue the phenotype of *Δyfh1* in response to oxidative stress

**Figure 3:**
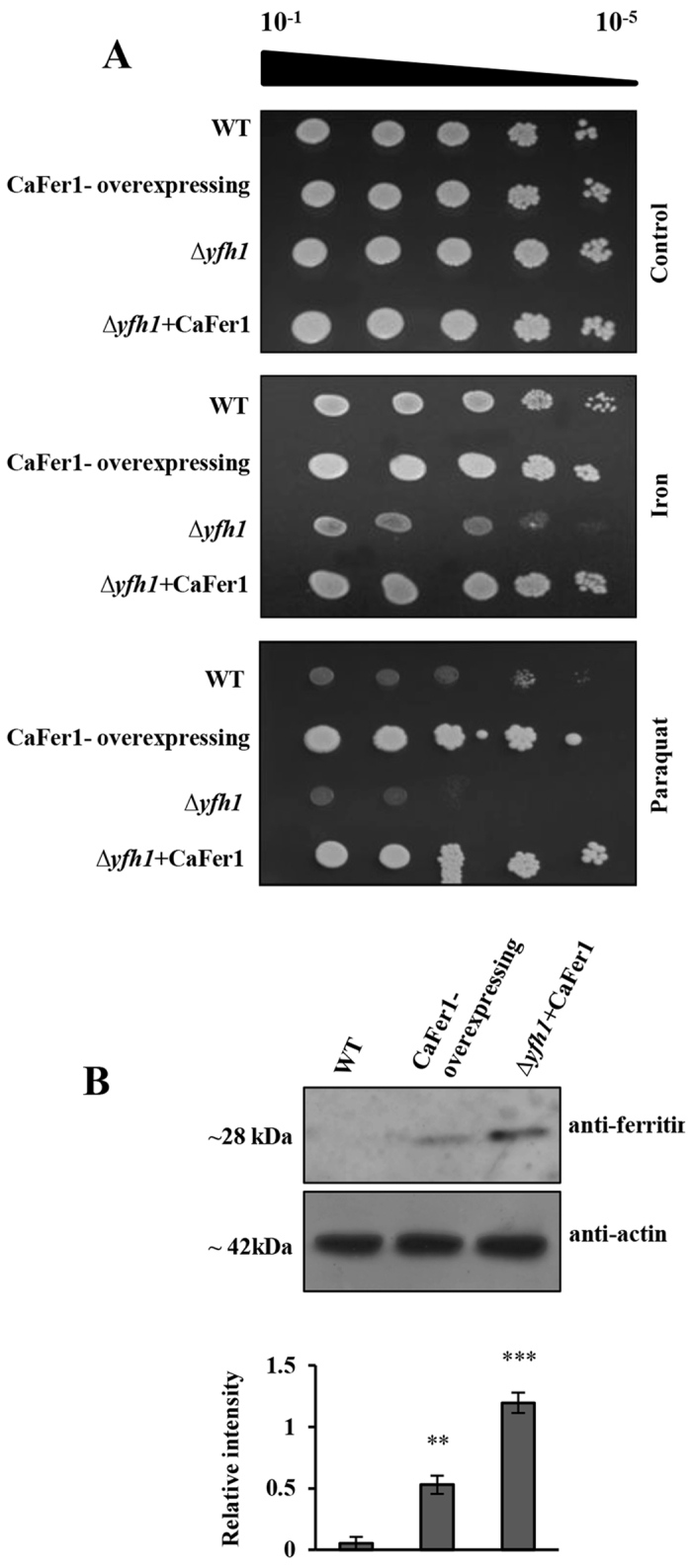
CaFer1 complements the function of *YFH1* in yeast. (**A**) Growth phenotype of WT, CaFer1-overexpressing, mutant (*Δyfh1*) and complemented (*Δyfh1* + *CaFer1*) strains in presence of 1 mM Fe and paraquat, in serial dilutions (10^−1^ to 10^−5^). (**B**) Immunoblot analysis and detection of CaFer1 in the CaFer1-overexpressing and complemented strains. Untransformed WT used as negative control. β-actin served as loading control. The bar graphs indicate the fold-expression as determined by band intensity of CaFer1. Error bars represent the SE from three replicates. Asterisks indicate significant differences relative to the WT background (Student’s t test). WT, wild-type.

Frataxin is a Fe storage protein that mainly participates in assembly of iron-sulphur cluster complex^31^, and is involved in oxidative stress responses. To investigate the role of CaFer1 in oxidative stress tolerance, we tested whether it could rescue the phenotype of yeast frataxin mutant, *Δyfh1* in oxidative stress condition. *YFH1* lacking yeast strains exhibit an increased mitochondrial and cellular Fe content resulting in hypersensitivity to oxidative stress. The mutant exhibits severe growth defects and increased sensitivity to oxidants. We monitored the growth of wild-type (WT), CaFer1*-*overexpressing, *Δyfh1* and complemented (*Δyfh1* + *CaFer1*) strains in presence of 1 mM Fe and paraquat independently, which induces oxidative stress (Fig. 3A). The complemented and overexpressing strains displayed better growth and enhanced resistance than mutant and/or WT. Furthermore, immunoblot analysis of complemented and overexpressing strains displayed higher accumulation of CaFer1 in complemented strain (Fig. 3B). These results indicate that CaFer1 can rescue the phenotype of frataxin mutant, presumably by increasing the storage of free iron molecules and decreasing the amount of ROS.

### CaFer1-overexpressing transgenic plants showed increased growth and development

**Figure 4:**
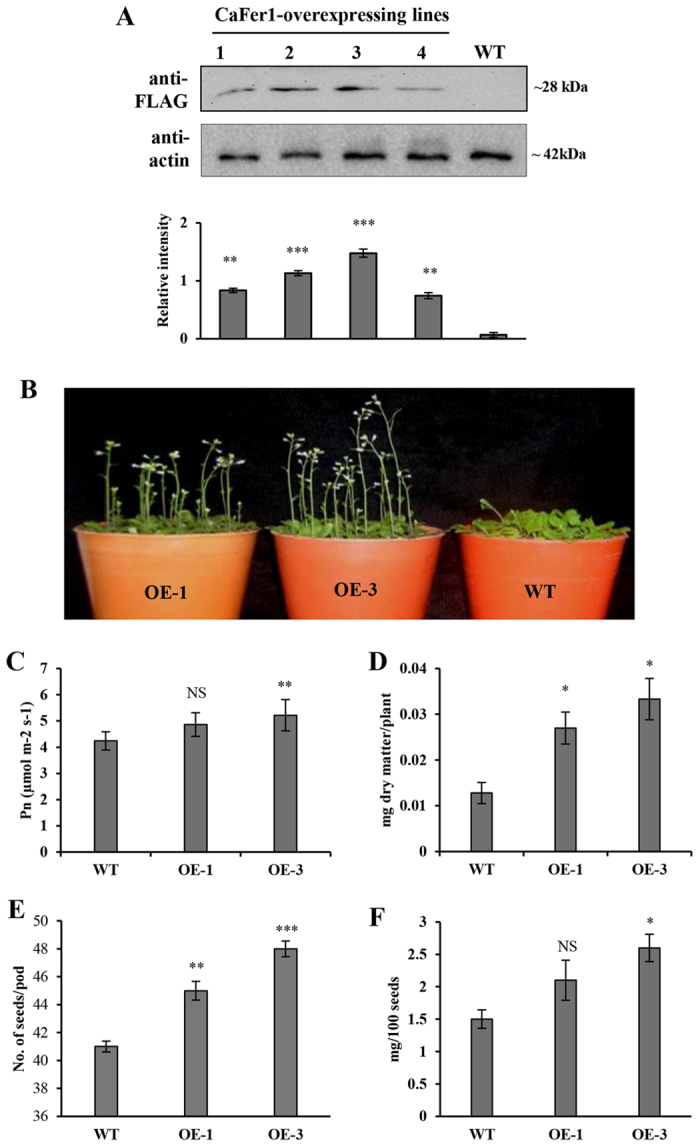
Physiological characterization of CaFer1-overexpressing seedlings. (**A**) Screening of transgene expression by immunoblot analysis using anti-FLAG antibody. Actin served as loading control. The bar graph indicates the fold-expression as determined by band intensity of CaFer1. (**B**) Morphometric analysis of developing seedlings of WT and in two OE lines, OE-1 and OE-3. (**C**) Enhanced net photosynthetic rates in OE-3 plants. (**D**) Enhanced biomass production in OE-3 plants. (**E**) Increased seed number and (**F**) increased seed weight in OE-3 plants as compared to WT. Error bars represent the SE from three replicates. Asterisks indicate significant differences relative to the WT background (Student’s t test). NS, not significant; WT, wild-type; OE, CaFer1-overexpressing.

Chickpea is highly recalcitrant to *in vitro* regeneration and genetic transformation^32^. Therefore, we made transgenic *Arabidopsis* by placing FLAG-tagged *CaFer1* cDNA under the control of *CAMV 35S* promoter (Supplementary Fig. 3A) and investigated the functional role of CaFer1. *CaFer1* integration was checked in different transgenic lines through PCR analysis (Supplementary Fig. 3B). No phenotypic abnormality, in any of the transgenic lines, was observed throughout their life cycle. On the basis of CaFer1 expression (Fig. 4A), two independent transgenic lines from T3 generation, one with moderate (OE-1) and the other with higher (OE-3) expression, were studied (Fig. 4B). The transgenic plants overexpressing CaFer1 had a higher photosynthetic rate (Fig. 4C) and 4-week-old transgenic seedlings showed increased biomass as compared to WT counterparts (Fig. 4D). It is likely that CaFer1 could provide enough Fe to the plant for photosynthesis leading to increased biomass production. Further, the overexpressed seedlings exhibited higher seed number (Fig. 4E) and seed weight (Fig. 4F), which could be due to increased silique length. OE-3 plants grew significantly faster than the WT plants; when OE-3 plants were at the pod stage, the WT plants were still in flowering stage (Supplementary Fig. 4A). Transgenic plants also showed a marked increase in pod size (Supplementary Fig. 4B). The OE-3 seedlings were better in growth phenotype and the physiological parameters than the OE-1 seedlings, henceforth OE-3 line referred to as OE was selected for further characterization. These results suggest the role of CaFer1 in plant growth and development.

### CaFer1-overexpressing plants showed increased stress tolerance

**Figure 5:**
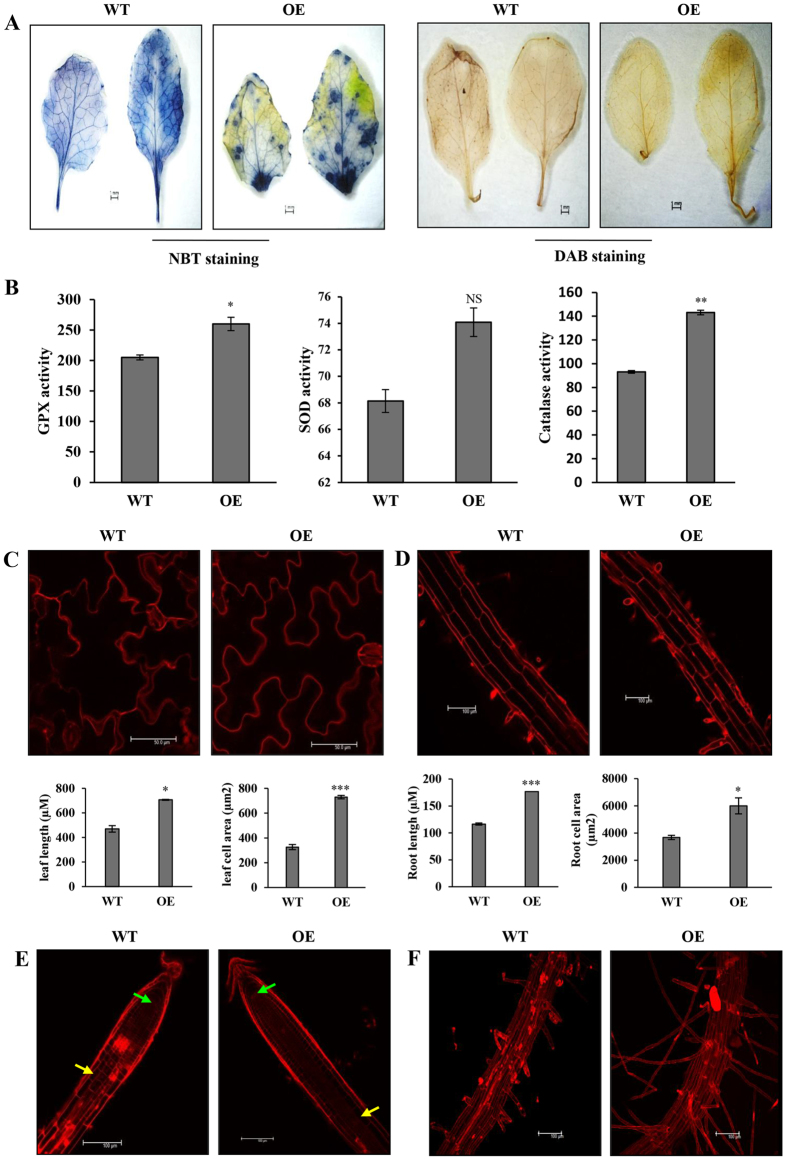
ROS accumulation and determination of increased cell number and cell size in CaFer1-overexpressing seedlings. (**A**) Leaves of WT and OE plants were stained with NBT and DAB dyes. (**B**) The histogram shows the activity of enzymes viz., GPX, SOD and catalase in OE and WT seedlings. (**C**) Representative figure and histogram displaying increased leaf cell size and area in WT and OE seedlings. The leaf, root and cell size were determined by ImageJ tool. (**D**) Representative image and histogram showing increased root cell size and area in WT and OE seedlings. Error bars represent the SE from three replicates. (**E**) Difference in cortical meristematic cells between the quiescent center (green arrows) and the transition zone (yellow arrows) in WT and OE seedlings. (**F**) Enhanced root hair density in OE seedlings. Comparative analysis was carried out with tissues from 8- to 10-day-old *Arabidopsis* seedlings stained with propidium iodide and imaged by confocal microscope. Experiments were performed in triplicates and images of one representative experiment are shown. Asterisks and indicate significant differences relative to the WT background (Student’s t test). NS, not significant; WT, wild-type; OE, CaFer1-overexpressing.

Next, we investigated the role of CaFer1 as an antioxidant in terms of limiting ROS production in 4-week-old WT and OE seedlings using nitro blue tetrazolium chloride (NBT), for superoxide anion, and 3, 3′-diaminobenzidine (DAB), for H_2_O_2_ detection. The transgenic leaves were less stained (Fig. 5A), which could be due to CaFer1-mediated reduction of ROS. Additionally, we examined the antioxidant machinery in CaFer1-overexpressing seedlings by analysing the activity of the enzymes involved viz., glutathione peroxidase (GPX), superoxide dismutase (SOD) and catalase. These enzymes displayed an increase in activity in the OE seedlings when compared to WT (Fig. 5B).

It has been previously reported that Fe-homeostasis and ROS distribution affect the root growth and differentiation, and Fe-overload reduces primary and lateral root length^33^. We, therefore, sought to know if CaFer1 overexpression affects the root architecture and differentiation along with shoot growth. Larger leaf cell size was observed in OE seedlings (Fig. 5C) when compared with WT. Analysis of root architecture revealed larger cells and increased root cell area (Fig. 5D). The OE seedlings also showed increased cell division (Fig. 5E) as well as increased number of root hairs (Fig. 5F). These results suggest that CaFer1 promotes increased root and shoot length possibly by regulating Fe-homeostasis and ROS production.

**Figure 6:**
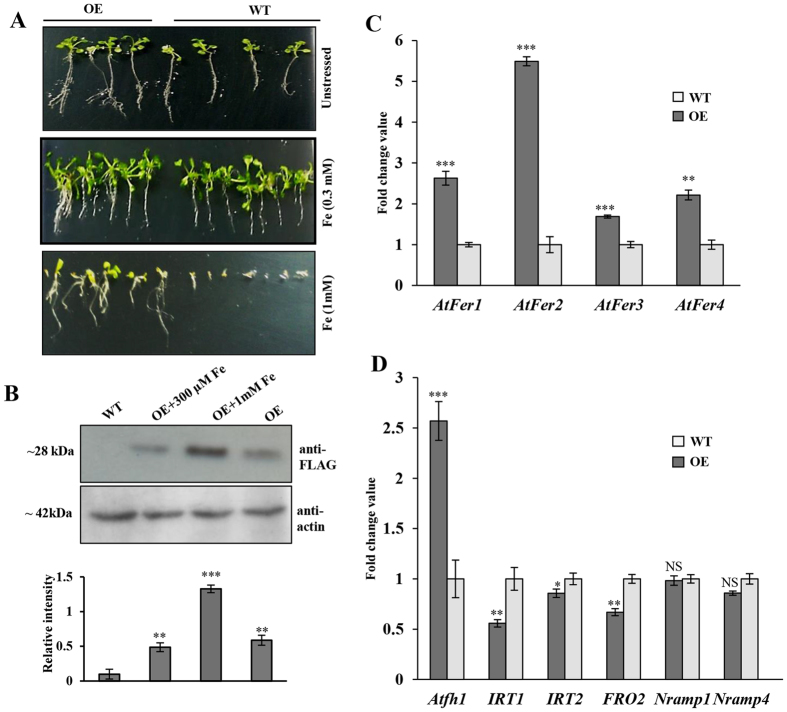
Iron-responsive phenotypes of CaFer1-overexpressing plants, and expression analysis of iron-responsive genes. (**A**) WT and OE seedlings were grown on MS media supplemented with different concentration of Fe. Plate assays were done in, at least, four replicates and results of one representative experiment is shown. (**B**) CaFer1 expression in OE seedlings treated with 300 μM and 1 mM Fe when compared with untreated OE and WT seedlings. Actin served as loading control. The bar graphs indicate the fold-expression as determined by band intensity of CaFer1. (**C**) Expression of ferritin genes (*AtFer1*–*4*) in OE and WT seedlings as determined by qRT-PCR. (**D**) Expression analysis of Fe-responsive genes in OE and WT seedlings. The expression levels of genes were normalized using the signal from the actin or ubiquitin genes. Error bars represent the SE from three replicates. Asterisks indicate significant differences relative to the WT (Student’s t test). NS, not significant; WT, wild-type; OE, CaFer1-overexpressing.

To know the iron-responsive function of CaFer1, we also examined the response of the OE seedlings to moderate (300 μM) and higher (1 mM) concentration of Fe. CaFer1-overexpressing seedlings did not show any noticeable difference in growth phenotype when compared to WT at moderate Fe concentration (Fig. 6A). The growth phenotype of OE seedlings at higher Fe concentration showed better growth when compared to WT (Fig. 6A). Immunoblot analysis revealed the induction of CaFer1 in presence of 1 mM Fe when compared to 300 μM Fe in OE seedlings (Fig. 6B). The unaltered expression of CaFer1 at 300 μM Fe (Figs 2C and 6B) could be the possible reason of unchanged phenotype of CaFer1-overexpressing seedlings at this concentration (Fig. 6A). The increased growth phenotype of OE seedlings at 1 mM Fe (Fig. 6A) can be correlated with its increased expression (Figs 2C and 6B). These results suggest that CaFer1 is responsible for Fe-mediated oxidative stress tolerance and is differentially regulated at distinct iron conditions. We also examined the phenotypic response of the OE seedlings to oxidative stressor, H_2_O_2_ (Supplementary Fig. 4C). The OE seedlings displayed better growth phenotype in presence of 10 mM H_2_O_2_ showing an increase of 2.5-fold in the average leaf area and root size during early-vegetative phase (15–21 d).

Further, the concentration of Fe along with other metals was also investigated in the OE plants (Supplementary Fig. 5). The energy-dispersive X-ray fluorescence spectrometry (EDXRF) analysis showed increased K and Cl level, but no significant change in Fe, S, P, and Ca level. Further, the OE plants exhibited low level of toxic metals, viz., Zn, Mn, Si and Cu, indicating a specific mechanism to cope with adverse growth conditions. These results suggest that CaFer1 might play an important role in maintaining metal ion homeostasis.

The analysis of other Fe-responsive genes in OE seedlings, displayed increased expression of iron storage genes, ferritins (*AtFer1-4*) and frataxin (*Atfh1*) as shown in Fig. 6C,D. In contrast, expression of Fe-deficiency responsive genes, viz., *IRT1*, *IRT2*, *FRO2*, *NRAMP1*, and *NRAMP4* either decreased or remained unaltered (Fig. 6D). It has earlier been reported that soybean ferritin overaccumulation in transgenic tobacco leads to an illegitimate Fe-sequestration due to which plants behave as Fe-deficient and consequently activate iron transport system^34^. CaFer1-overexpressing plants showed no Fe-deficient phenotype, which could be a possible reason for decreased expression of Fe-deficiency responsive genes as they are induced only in Fe-deficiency. The increased expression of iron storage genes (ferritins and frataxin) in OE seedlings explains the reduced production of ROS vis-a-vis limiting oxidative stress, which could be due to excess availability of free Fe radicals.

### Compartmentalization of CaFer1

**Figure 7:**
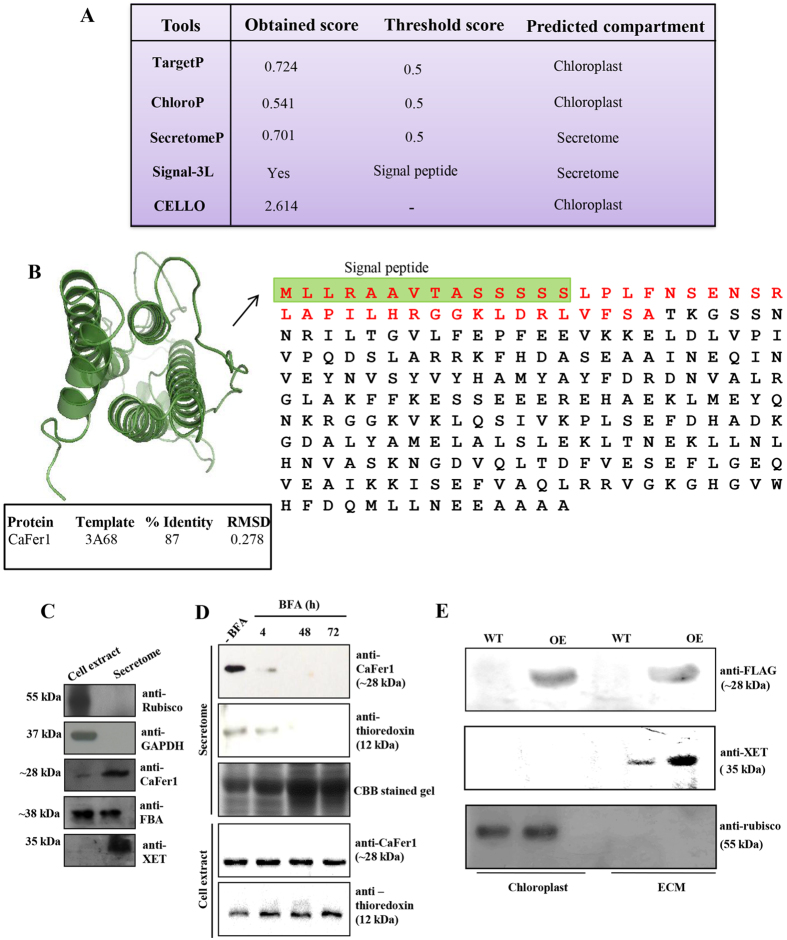
Compartmentalization of CaFer1. (**A**) Bioinformatics prediction of CaFer1 to the chloroplast and the secretome. (**B**) The model of CaFer1 generated by comparative modeling, shown in ribbon view (right upper panel). The percent identity of modeled structure of CaFer1 with the template, and the RMSD values were given in the table (right lower panel). The non-conserved amino acid sequence of CaFer1 (red color) from that of other phytoferritins with predicted signal peptide, is shown in left panel. (**C**) Purity assessment and immunodetection of CaFer1, FBA and XET in the chickpea secretome. Immunoreactions with Rubisco and GAPDH served as negative controls for secretome. (**D**) BFA-mediated inhibition of CaFer1 and thioredoxin transport. ‘−BFA’ represents the untreated secretome. (**E**) CaFer1 was detected in extracellular space and chloroplast of OE plants with anti-FLAG antibody. XET was used as positive control for extracellular space and rubisco for chloroplast. WT, wild-type; OE, CaFer1-overexpressing.

Phytoferritins are generally localized to the plastids and also reported to the mitochondria. To examine the subcellular location of CaFer1, we used TargetP (http://www.cbs.dtu.dk/services/TargetP/) and CELLO v.2.5, which predict multiple cellular compartmentalization, ChloroP (http://www.cbs.dtu.dk/services/ChloroP/) for chloroplast, Secretome 2.0 (http://www.cbs.dtu.dk/services/SecretomeP/) for secreted protein and SIGNAL-3L (www.csbio.sjtu.edu.cn/bioinf/Signal-3L/) for prediction of signal peptide. The analysis showed possible localization of CaFer1 to the chloroplast and secretome but not in the mitochondria (Fig. 7A). CaFer1 sequence analysis revealed 79% amino acid sequence similarity with other phytoferritins, except for the nonconserved N-terminal region (42 amino acid), unique to CaFer1 (Fig. 7B, left and right panel). The modeled structure of CaFer1, generated using MODELLER 9.10, exhibited significant structural similarity with the *Glycine max* ferritin template, with low RMSD value (Fig. 7B, left upper and lower panel), confirming the quality of the model. CaFer1 contains signal peptide (1–14 amino acids, green box) preceded with transit peptide (41 amino acids, red sequence), which again predicted its secreted nature along with its chloroplast localization (Fig. 7B, right panel).

Our previous study indicated the presence of ferritin in the ECM^25^ and in secretome of chickpea^35^, which prompted us to explore the secreted nature of ferritin/s. As phytoferritins have never been characterized in this compartment, we next examined whether ferritin is a bona fide constituent of the secretome. The purity of the callus culture derived secretome was validated by immunoblot analysis using anti-rubisco and anti-GAPDH antibodies. The detection of rubisco and GAPDH in the cell extract and not in the secretome confirms the purity of the fraction. The purity was further examined by two known secreted proteins, FBA (fructose 1,6 bisphosphate aldolase) and XET (xyloglucan endotransglycosylase). While FBA was detected in the cell extract and secretome, XET only in the secretome, but not in the cell extract (Fig. 7C). Immunoblot analysis could detect CaFer1 in the chickpea secretome as well as in the cell extracts (Fig. 7C) using anti-CaFer1 antibody. To further check if the secretion of chickpea ferritin is merely a result of cell lysis, the cell suspension culture was treated with brefeldin A (BFA, 10 μg/ml), an inhibitor of secreted proteins. Secretion of ferritin was inhibited after 4 h of treatment and gradually disappeared, whereas no inhibition was observed in the untreated secretome and cell extract. Secretory thioredoxin was used as a positive control, which showed a similar expression pattern (Fig. 7D). These results strongly suggest the active secretion of ferritin to the extracellular space, and not a by-product of passive release due to cell lysis. To further validate the result, we used the protein extracts from FLAG-tagged CaFer1-overexpressing (OE) and WT seedlings. Immunoblot analysis was carried out with anti-FLAG antibody using purified ECM^36^ and chloroplast fraction^37^ from OE seedlings. The results indicate the presence of FLAG-tagged CaFer1 in the ECM and chloroplast in OE seedlings (Fig. 7E). We used the ECM specific protein, XET and chloroplast specific protein, rubisco as positive controls. While XET was exclusively present in the ECM, rubisco was detected in the chloroplast (Fig. 7E), which confirms the purity of the fractions examined. These results suggest that CaFer1 is a resident of chloroplast which might also be secreted to the ECM. It is possible that plastid-localized CaFer1 would be transported through the secretory pathway as previously reported for α-carbonic anhydrase^38^ and/or it is dually localized.

To further investigate the Fe-responsive function of secreted ferritin and role in oxidative stress, we quantified the expression using immunoblot analysis. The treatment with moderate Fe concentration (300 μM) did not show any cell death, but higher Fe concentration (1 mM) was toxic to the cells (Supplementary Fig. 6A). The immunoblot analysis showed low abundance of ferritin at moderate concentration, while upregulated expression at high iron concentration, albeit less than that in the untreated control. However, the treatment with 10 mM H_2_O_2_ led to the maximum expression (Supplementary Fig. 6B).

## Discussion

The transcription of ferritin in plants is induced by myriad of environmental factors, which then stimulates ROS production^3^. Our results suggest the developmental regulation of *CaFer1* and its involvement in multivariate abiotic stresses, besides ABA response. The differential accumulation of *CaFer1* in chickpea challenged with Foc suggests that in addition to abiotic stresses, it might be involved in biotic stresses. It is to be noted that *CaFer1* was isolated from chickpea cv. JG-62, which is hypersensitive to Foc infection^39^. We therefore propose that CaFer1 may function as an antioxidant, which is downregulated at the onset of infection, similar to other antioxidants^40^, but highly upregulated during disease progression to prevent oxidative damage due to higher accumulation of ROS. The role of CaFer1 in other stresses could also correlate with its antioxidant nature, as ROS can be produced via different stresses.

To establish the function of CaFer1 in oxidative stress response, we performed complementation assay in yeast frataxin mutant, *Δyfh1*. Excess Fe-accumulation in the mitochondria of *Δyfh1* leads to loss of mitochondrial DNA, nuclear damage, and generation of high levels of hydrogen peroxide^41^. This eventually increases sensitivity to oxidants and reduces growth rate. Human cytoplasmic ferritin complements *Δyfh1* and improves cell growth in yeast^42^. CaFer1 could rescue the phenotype of *Δyfh1*, and impart improved growth. The increased accumulation of CaFer1 in complemented strain suggests that CaFer1 could behave more actively in the absence of major yeast iron-storage protein, frataxin to overcome the effect of ROS. Intriguingly, frataxin is a mitochondrial resident protein^43^, and CaFer1 is not localized to the mitochondria, yet complements frataxin function. It seems that CaFer1 might prevent excess accumulation of ROS by scavenging Fe, which leads to less Fe-migration to the mitochondria. It is also likely that cells grown in Fe-deficient conditions might accumulate less Fe in the mitochondria, thus minimizing oxidative damage. The role of CaFer1 in oxidative stress was further validated in OE plants where it could minimize the production of ROS.

Ferritins are responsible for maintaining iron homeostasis and detoxifying iron overload. Several studies indicate that iron overload leads to an abnormally high level of Fe and results in increased ROS production and elevation of oxidative stress responses^3^. The connection between Fe and ROS has also been reported in *Arabidopsis* mutants for ferritin^15^. In plants, ferritin is repressed under low Fe-supply or Fe-deprivation, and plays a key role in oxidative stress tolerance. Iron strongly induces expression of phytoferritins^5^,^20^,^28^,^44^. In this study, *CaFer1* transcript expression was induced in response to iron overload, while it remained unaltered when treated with moderate Fe concentration, implying a distinct mechanism of *CaFer1* accumulation at different iron conditions, which needs to be investigated further. However, the protein accumulation presented a differential expression pattern from the transcript level in response to iron and H_2_O_2_, which suggests possible post-transcriptional regulation of CaFer1.

The previous reports from transgenic plants over-expressing ferritins revealed the relations between ferritin and ROS management, besides its involvement in the protection against oxidative stress upon photoinhibition^19^,^30^,^45^,^34^. Consistent with such observations, CaFer1-overexpressing seedlings showed better growth and development, and its involvement in oxidative stress and iron buffering.

In plants, ferritins have been reported in chloroplasts^46^ and mitochondria^47^, while most animal ferritins are cytosolic iron-storage proteins though they have also been detected extracellularly^23^. In *Arabidopsis*, one of the ferritins, FER4, is dually localized in mitochondria and chloroplasts^48^. Bioinformatic analysis did not indicate CaFer1 localization in mitochondria, but predicted to be localized to the chloroplast and secretome. Many organisms show secretion of extracellular iron-binding molecules, such as transferrins or siderophores, in response to Fe-deprivation. However, CaFer1 does not show sequence similarity to these proteins, but may have an analogous function like the secreted proteins, FEA1 and FEA2^21^. Since ferritin is encoded by a multigene family, some of the genes may encode functionally distinct proteins. The localization of CaFer1 to the chloroplast and ECM indicates its distinct nature as compared to other phytoferritins. It is well established that ROS is a by-product of a multitude of stresses. ECM, being the first defense system of the cell, needs to limit the ROS so generated and maintain the redox balance. The localization of CaFer1 to this compartment might help maintain the level of ROS thus, contributing to homeostasis. The findings of this study reassess the role of ferritin and suggest that CaFer1 may participate in an alternative mechanism for Fe response and stress tolerance in plants. Hence, the multifunctional *CaFer1* seems promising as a prime candidate gene for improved growth and development, stress tolerance and Fe-equilibrium.

## Methods

### Plant growth conditions and stress treatments

The chickpea seedlings were grown in pots containing a mixture of soil and soilrite (2:1, w/w), and maintained at 25 ± 2 °C with 50 ± 5% relative humidity under 16 h photoperiod (300 μmol m^−2^ s^−1^ light intensity). Dehydration was imposed on 3-week-old seedlings by withdrawing water for up to 192 h. The stressed seedlings were then re-watered allowing complete recovery of the stressed seedlings for 24 h (R-24). For hypersalinity treatments, seedlings were supplemented with half-strength Hoagland’s medium for three weeks, followed by treatment with different concentrations (100, 250 and 500 mM) of NaCl. The seedlings were also sprayed with ABA (100 μM). To study the effect of Fe, MS media was supplemented with ferric citrate (300 μM and 1 mM). Fungal infection was inflicted with a fresh culture of *Fusarium oxysporum* f. sp. *ciceris* (Foc) obtained from a monoconidal culture. The cultures were filtered through eight layers of sterile cheesecloth to remove mycelial mats. The filtrate was pelleted by centrifugation (10,000 × g), washed three times to remove all traces of nutrients, and diluted to an OD_600_ of 10^6^ spores/ml. The seedlings grown on MS media were carefully removed, washed free of media with distilled water and infected with the fungal inoculum by dipping the root in the culture till the desired time points.

*Arabidopsis* seedlings were grown in growth chamber and maintained at 18–21 °C with 60–70% relative humidity, under 16 h photoperiod (270 μmol m^−2^ s^−1^ light intensity). To assess the stress-responsive growth phenotype of CaFer1-overexpressing (OE) seedlings, sterilized seeds were kept in parallel onto identical plates supplemented with Fe (300 μM and 1 mM) and 10 mM H_2_O_2_. Potted *Arabidopsis* plants were grown for 4 weeks, and seedlings were grown for 8 days to 2 weeks. The tissues, harvested at various time intervals, were instantly frozen in liquid nitrogen and stored at −80 °C, unless stated otherwise.

### Immunodetection

1-DE was performed by resolving the protein on a uniform 12.5% gel and then electrotransferring to nitrocellulose membranes at 150 mA for 2 h. The membranes were subsequently blocked with 5% (w/v) non-fat milk for 1 h at 25 °C. The membranes were probed with anti-FLAG (F1804; Sigma), anti-actin (A0480; Sigma) and anti-ferritin (ab7332; abcam), anti-CaFer1 (customized against 54–66 (GVLFEPFEEVKKE) amino acid sequence, anti-FBA (AS08294; Agrisera) and anti-thioredoxin (T0803; Sigma-Aldrich) antibodies diluted to 1:1000 in Tris-buffered saline (TBS). The antibody bound proteins were detected by incubation with horseradish peroxidase-conjugated anti-rabbit IgG, and the bands were visualized using the ECL detection system (GE Healthcare).

### Screening of cDNA library and cloning of *CaFer1*

The 3′ RACE PCR was performed with 1 μg total RNA using a 3′ RACE kit (Invitrogen). For cDNA synthesis, RNA was primed with oligo (dT) primer, and amplification was done with reverse transcriptase. PCR amplification was done with a gene-specific primer 5′-GAAGAAAGAGAGCATGCT-3′, designed against one of the sequence tags identified by MS/MS analysis for ferritin and the universal adaptor primer (UAP) as per the manufacturer’s instructions. The partial 0.6 kb clone thus obtained was subsequently cloned into pGEM-T Easy vector (Promega) and confirmed by sequencing. A chickpea cDNA library was constructed using SMART cDNA library construction kit (Clontech) with RNA prepared from unstressed and stressed seedlings. Plaque lifts of the library on nylon membranes were screened by the partial clone as probe following standard procedure^49^. The selected clones were purified and confirmed by sequencing with the ABIPRISM 3700 DNA Analyzer (Applied Biosystems, USA).

### *In silico* analyses

Computer-assisted sequence analysis was carried out and a phylogenetic tree was constructed for CaFer1 from an amino acid alignment using the default parameters (http://www.ebi.uk/Tool/clustalw/). Multiple alignment of amino acids was performed with ClustalW program, and the phylogram was obtained using MEGA version 5^50^. The protein models for CaFer1 was generated using comparative modeling approach with MODELLER 9.10^51^ programs in Linux environment. The Glycine max ferritin (PDB ID A368) was taken as template on the basis of NCBI PDB-BLAST result. The model with best DOPE score was selected for the final study. The modeled protein exhibited significant structural similarity with the template, confirming the quality of the modeled protein. The PyMOL (PyMOL Molecular Graphics System, Version 1.1 Schrodinger, LLC) was used to study and visualize the modeled proteins.

### Northern blot analysis

Total RNA was isolated either using the RNeasy Plant Mini kit (Qiagen) or the TriPure reagent (Invitrogen). RNA gel electrophoresis was performed as described^49^, blotted overnight onto a nylon membrane Hybond-N+ (Amersham Biosciences, UK), and fixed by UV cross-linking at 1200 j/cm for 30 s. The analyses were carried out with 15 μg RNA in each lane. Immobilized RNAs were hybridized with ^32^P-labeled probe generated from cDNA of *CaFer1*.

### Transcript analysis by qRT-PCR

Total RNAs were isolated from unstressed and stressed seedlings. The preparation of cDNA was accomplished using SuperScript^®^ VILO™ cDNA Synthesis Kit (Invitrogen). An aliquot of 2.5 μg RNA was used as initial sample for cDNA preparation, and diluted cDNA was used for RT-PCR. RT-PCR was performed with the ABI PRISM 7700 SDS (Applied Biosystems, USA) using SYBR Green PCR Master Mix (Brilliant III Ultra-Fast SYBR R, Green Q PCR master mix, Agilent technologies) in a final volume of 20 μl, including cDNA template (10–20 ng) and appropriate primer pairs (final concentration 900 nM) (Supplementary Table 1). The parameters were as follows: 10 min at 95 °C, and 40 cycles of 15 s at 95 °C and 1 min at 60 °C. Three technical and biological repeats were performed for each of the candidate genes. Ubiquitin, actin and EF1α were used as the internal standards.

### Complementation of yeast *Δyfh1* mutant by *CaFer1*

Wild-type yeast (FY1679-02B), and *Δyfh1* mutant (FKENO15-01A) strains were transformed with pAG426GPD-ccdB-EYFP vector with constitutive promoter containing the *CaFer1* ORF with stop codon (primers used are listed in Supplementary Table 1). Yeast transformation was performed using lithium acetate method (Geno Technology Inc.), and selected on SD-Ura (Invitrogen) by growing the cells at 30 °C for 3–4 days. The overnight grown cultures of the respective strains were diluted to OD_600_ of 0.1 in the YPD medium and allowed to grow till OD_600_ = 0.8. Thereafter, the serial dilutions [10^−1^ to 10^−5^] were prepared for each strain and plated. The plates were incubated at 30 °C for 2 days. The growth of WT, CaFer1-overexpressing, *Δyfh1*, and complemented strains was checked under normal conditions, as well as under 1 mM Fe- and paraquat-induced oxidative stress.

### Overexpression of CaFer1 in Arabidopsis and examination of stress tolerance

Binary vector pGWB411 was used as a Gateway destination vector^52^ for the expression of recombinant CaFer1 fused with C-terminal FLAG tag, under the control of 35S promoter. The *CaFer1* construct was first transformed into *Agrobacterium tumefaciens* (GV3101 cells), followed by transformation to *Arabidopsis* by floral dip method^53^. T1 populations were selected on MS plates supplemented with 50 mg/l kanamycin. The putative transgenic lines were confirmed by PCR (primers used are listed in Supplementary Table 1) and transcript analysis. Four independent T3 transgenic plants (OE-1 to OE-4) were selected on the basis of *CaFer1* expression.

### Measurement of photosynthesis

Net photosynthesis was measured using a portable Gas Exchange Fluorescence System (GFS-3000; Heinz Walz, Germany). The measurements were made under saturating irradiance (1000 μmol photons m^−2 ^s^−1^) and constant airflow of 750 μl/s at 25 °C. Five independent leaves, each from WT and OE seedlings were placed directly into the measuring cell (3 cm^2^) for the measurements.

### ROS detection

Leaves of WT and OE plants were immersed in solution containing 1 mg/ml DAB (pH 3.8) and NBT (pH 7.5) in 50 mM sodium phosphate, respectively and vacuum infiltrated. After staining with DAB and NBT, the leaves were bleached by incubating in boiled 95% ethanol. Stained leaves were visualized and photographed under bright field microscope.

### Development of callus culture and analysis

A homogenous chickpea suspension culture was developed from embryogenic calli^35^. The cultures were independently supplemented with 300 μM and 1 mM ferric citrate, and 10 mM H_2_O_2_. The viability of the suspension culture was examined microscopically using Evans blue staining.

### Determination of metal content

Concentration of metal ions in WT and CaFer1-overexpressing plants was determined by EDXRF analysis. Dry powders from the harvested tissues were used for the analysis. The measurements were performed on the EDXRF spectrometer (PANalytical, Netherland) with a Ge solid state detector.

### Enzyme assays

Tissue (0.5 g) was ground in liquid N_2_ to fine powder and then homogenized in 3.0 ml KPO_4_ (phosphate) buffer (pH 7.0). The lysate was then centrifuged at 16,000 × g for 20 min at 4 °C. The supernatant was used to measure the activity of SOD and catalase^54^, as well as the GPX activity^55^.

### Statistical analysis

One-class t-test was used to test the three means of the expression levels of each gene and the other physiological parameters among the control (untreated and wild-type plants), and treated and transgenic plants. The values p ≤ 0.001 were represented by ***p ≤ 0.01 as ** and p ≤ 0.05 as *p > 0.05 was considered as NS (not significant). Sigma plot version 12.0 was used for the t-test analysis.

## Additional Information

****Accession codes:**** *Arabidopsis* sequence data from this article can be found in the GeneBank/EMBL databases under the following gi numbers: AtIRT1, >gi|79478109:42-1085; AtIRT2, >gi|79325170:10-1062; NRAMP1, >gi|30699540:53-1651; NRAMP4, >gi|145359779:96-1634; FRO2,>gi|42561598|; Atfh1, >gi|145339957:15-578; AtFer1,>(gi|8163919:1481-1788; AtFer2, >gi|145338364:54-815; AtFer3, >gi|145339559:52-831; AtFer4, >gi|186506837:67-846. Chickpea sequences can be retrieved from Chickpea Transcriptome Database, FRO2, >TC16132; CaIRT1, >TC06693; NRAMP4, >TC07580; NRAMP1, >TC23658.

**How to cite this article**: Parveen, S. *et al.* Chickpea Ferritin CaFer1 Participates in Oxidative Stress Response, and Promotes Growth and Development. *Sci. Rep.* **6**, 31218; doi: 10.1038/srep31218 (2016).

## Supplementary Material

Supplementary Information
