# Supplementary material for: Chickpea Ferritin CaFer1 Participates in Oxidative Stress Response, and Promotes Growth and Development
Source: Sci Rep. 2016 Aug 9;6:31218. doi: 10.1038/srep31218 (PMC4977498; doi:10.1038/srep31218)
Supplement: Supplementary Information [file srep31218-s1.pdf]

Chickpea Ferritin CaFer1 Participates in Oxidative Stress Response, and Promotes Growth and Development

Shaista Parveen[1], Deepti Bhushan Gupta[2], Suchismita Dass[1], Amit Kumar[1], Aarti Pandey[1], Subhra Chakraborty[1] and Niranjan Chakraborty[1]

[1]National Institute of Plant Genome Research, Aruna Asaf Ali Marg, New Delhi-110067, India.

[2]Department of Biotechnology, TERI University, New Delhi.

Running title: CaFer1 participates in stress response

Address Correspondence:

¶Dr. Niranjan Chakraborty

National Institute for Plant Genome Research

Aruna Asaf Ali Marg, New Delhi-110067, India.

E-mail: nchakraborty@nipgr.ac.in

Tel: 00-91-11-26735178

Fax: 00-91-11-26741658

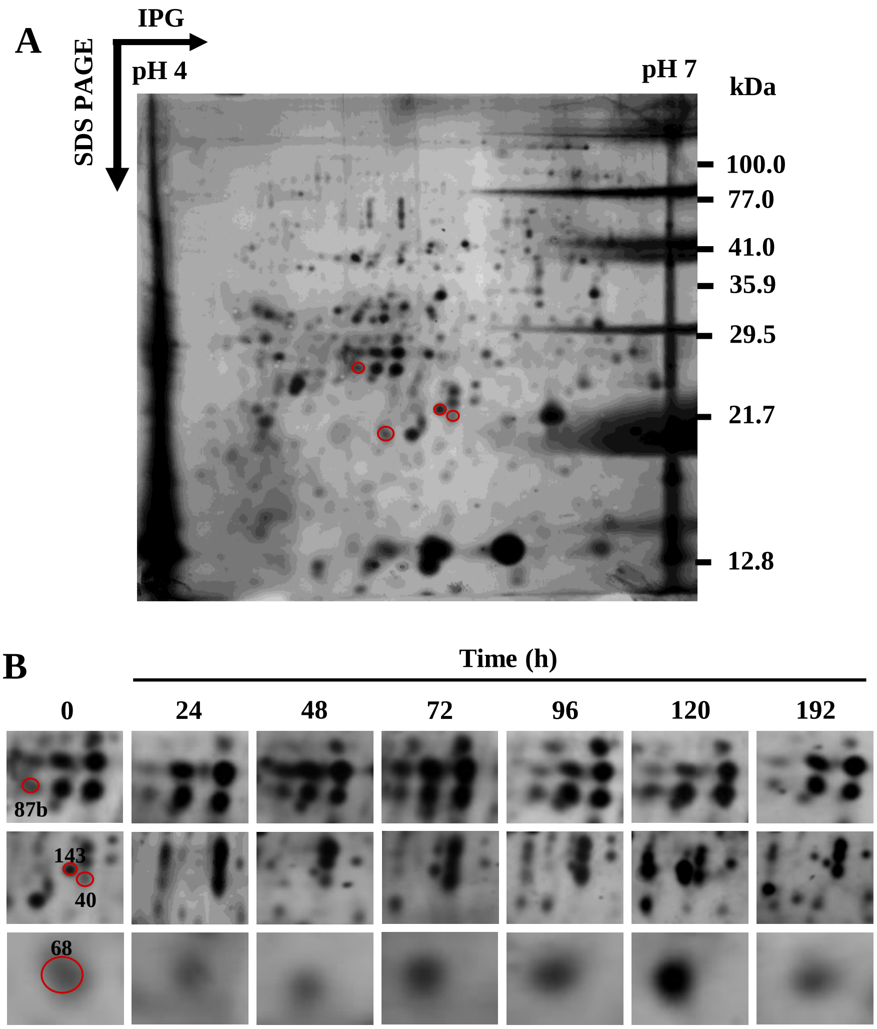

Supplementary Fig. 1. Screening of dehydration-responsive ECM proteome and identification of phytoferritins. (**A**) 2-DE profile of dehydration-responsive ECM proteome of chickpea. Differentially regulated spots of ferritins are encircled. (**B**) Zoomed-in gel sections correspond to the encircled regions in (**A**) showing temporal changes of ferritin proteins.

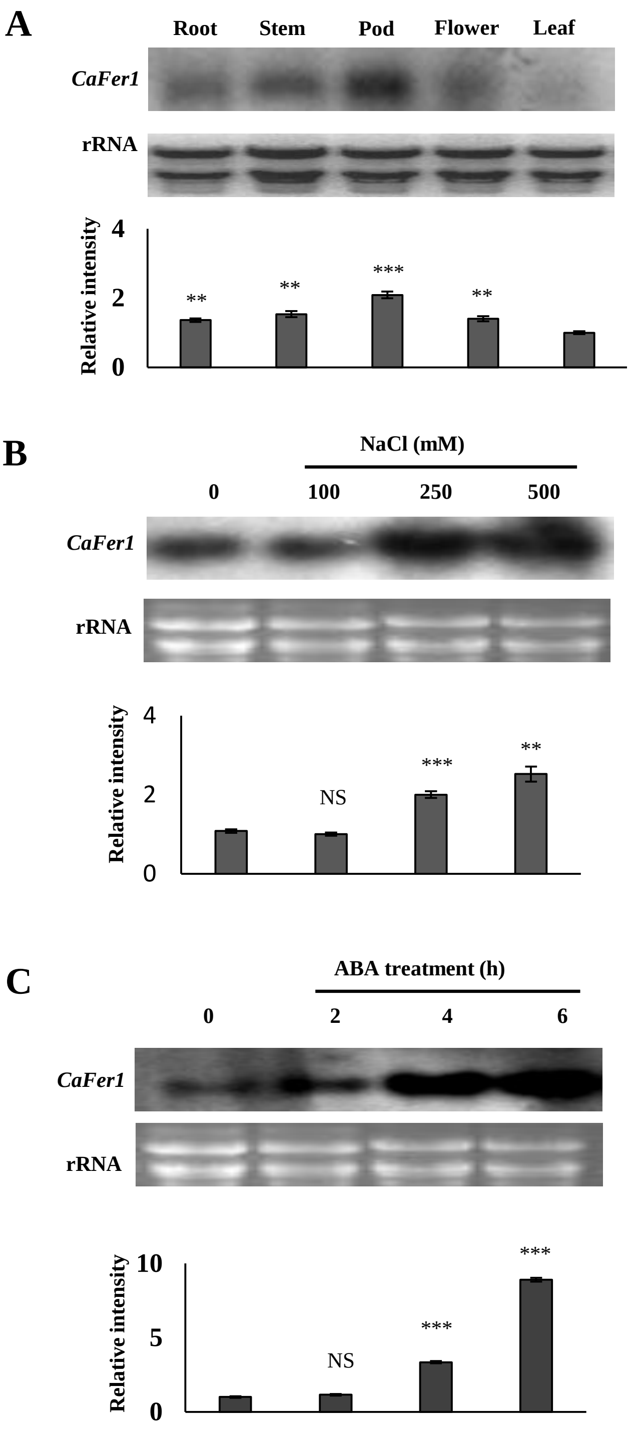

Supplementary Fig. 2. Expression analysis of *CaFer1* in different tissues, in response to hypersalinity and ABA treatment. (**A**) Northern blot showing expression of *CaFer1* in different tissues of 3-week-old chickpea plants. (**B**) Dose-dependent expression of *CaFer1* in response to salt. (**C**) Regulated expression of *CaFer1* in response to ABA treatment. The analyses were carried out with 15 µg total RNA in each lane. Blots were hybridized with ^{32}P-labeled 0.8 kb *CaFer1*. Ethidium bromide-stained rRNA served as loading control. Experiments were carried out in triplicates and the representative images are shown. Error bars represent the SE from three replicates. Asterisks indicate significant differences relative to the leaf and 0 h background (Student's t test). NS, not significant.

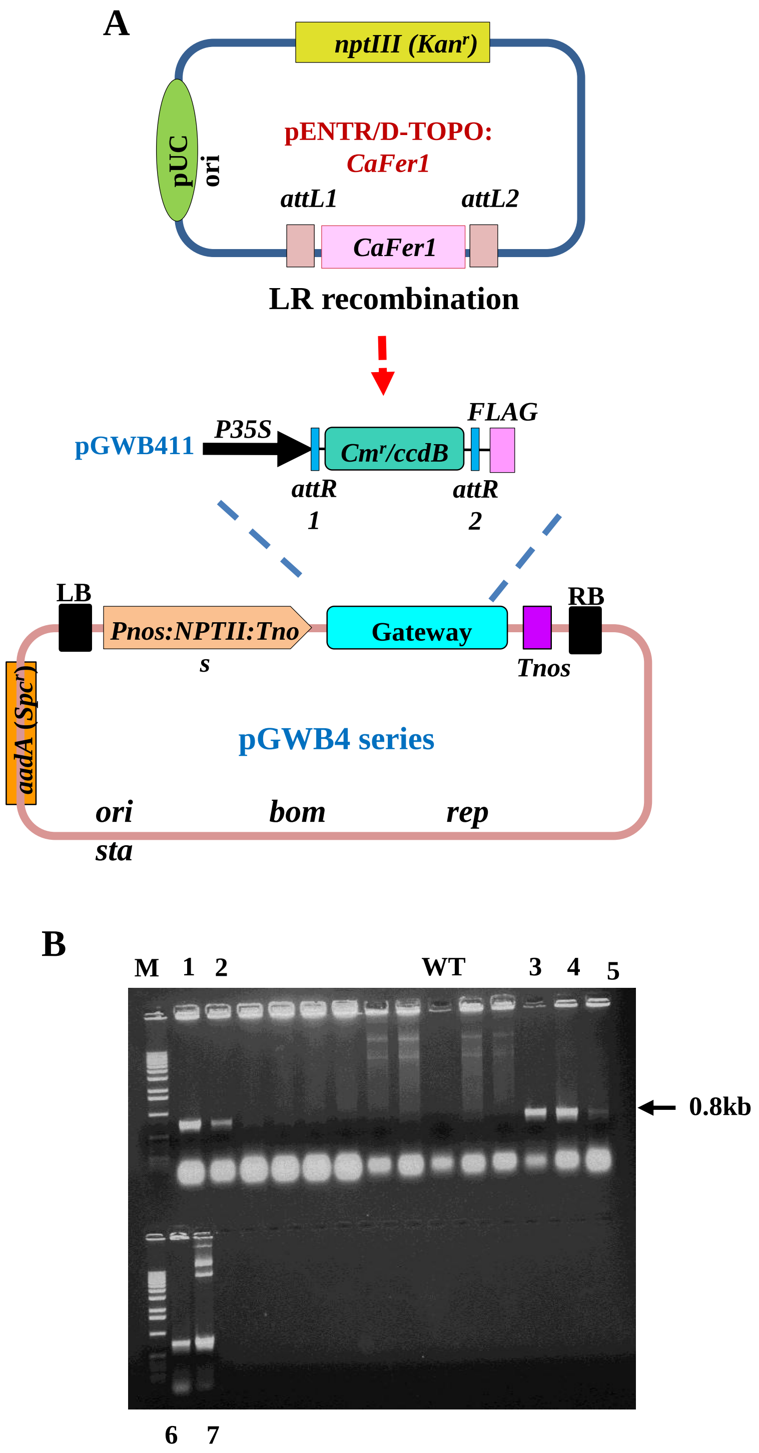

Supplementary Fig. 3. Cloning of *CaFer1* in plant-specific vector. (**A**) ORF of *CaFer1* was cloned in plant-specific gateway destination vector tagged with FLAG. (**B**) *CaFer1* insertion was checked by PCR in CaFer1-overexpressing *Arabidopsis* seedlings grown on kanamycin plates. M, marker; WT, wild-type; 1, 2, 3, 4, 5, 6 and 7 are the CaFer1-overexpressing lines.

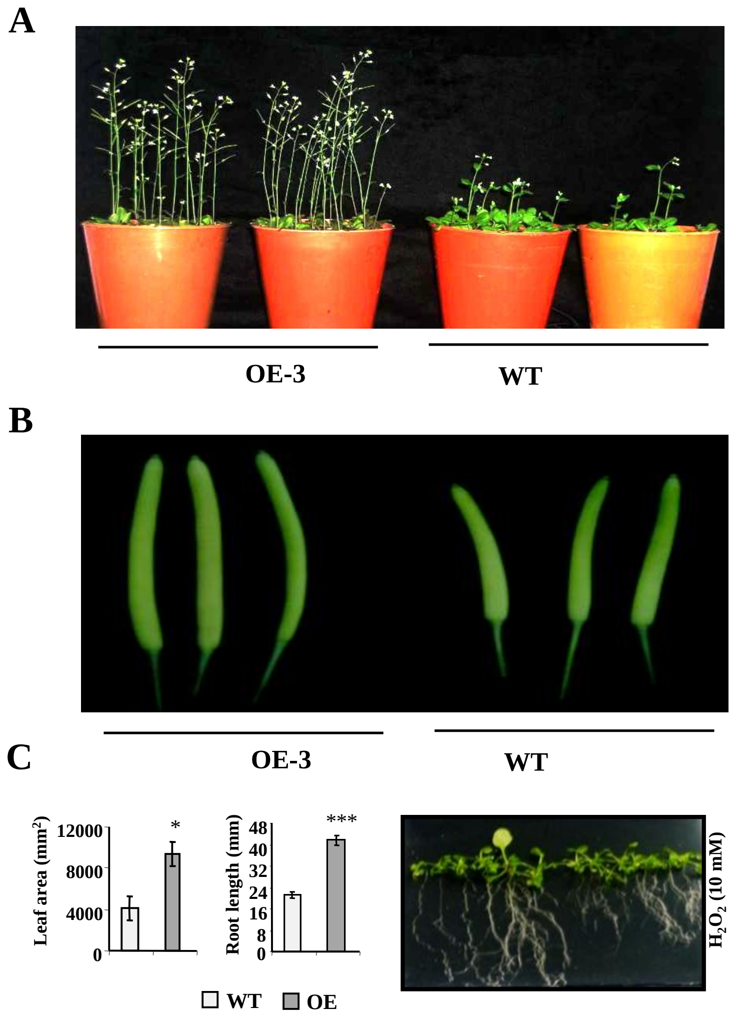

Supplementary Fig. 4. Physiological screening of OE-3 plants. (**A**) Growth phenotype of OE-3 line. (**B**) Increased pods in OE-3 plants. **(C)** WT and OE seedlings were grown on MS media supplemented with 10 mM H_2O_2. Histograms represent the leaf area and root length of the seedlings on the respective plate. Plate assay was done in, at least, four replicates and results of one representative experiment are shown. Error bars represent the SE from three replicates. Asterisks indicate significant differences relative to the WT background (Student’s t test). WT, wild-type; OE, CaFer1-overexpressing.

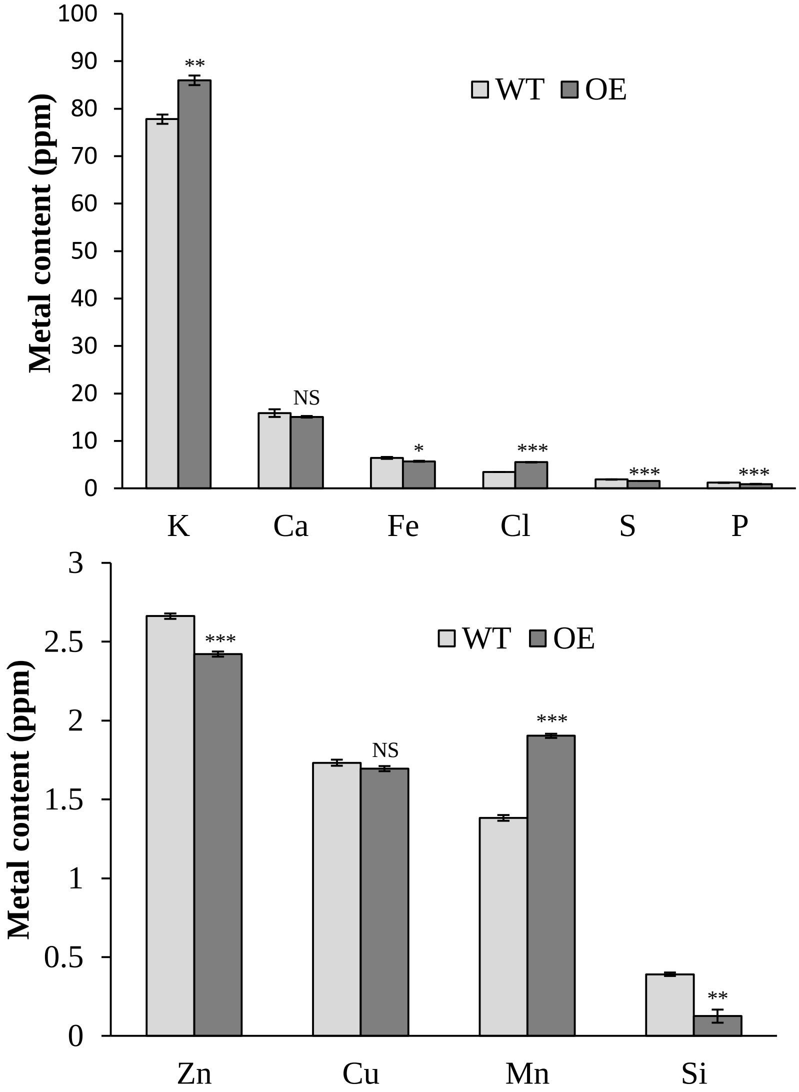

Supplementary Fig. 5. Comparison of metal content in WT and OE plants. Equal amount of tissue samples from WT and OE plants were dried at 105°C in an oven and grinded to fine powder. Metal contents were determined by EDXRF analysis. Error bars represent the SE from three biological replicates. Asterisks indicate significant differences relative to the WT background (Student's t test). NS, not significant; WT, wild-type; OE, CaFer1-overexpressing.

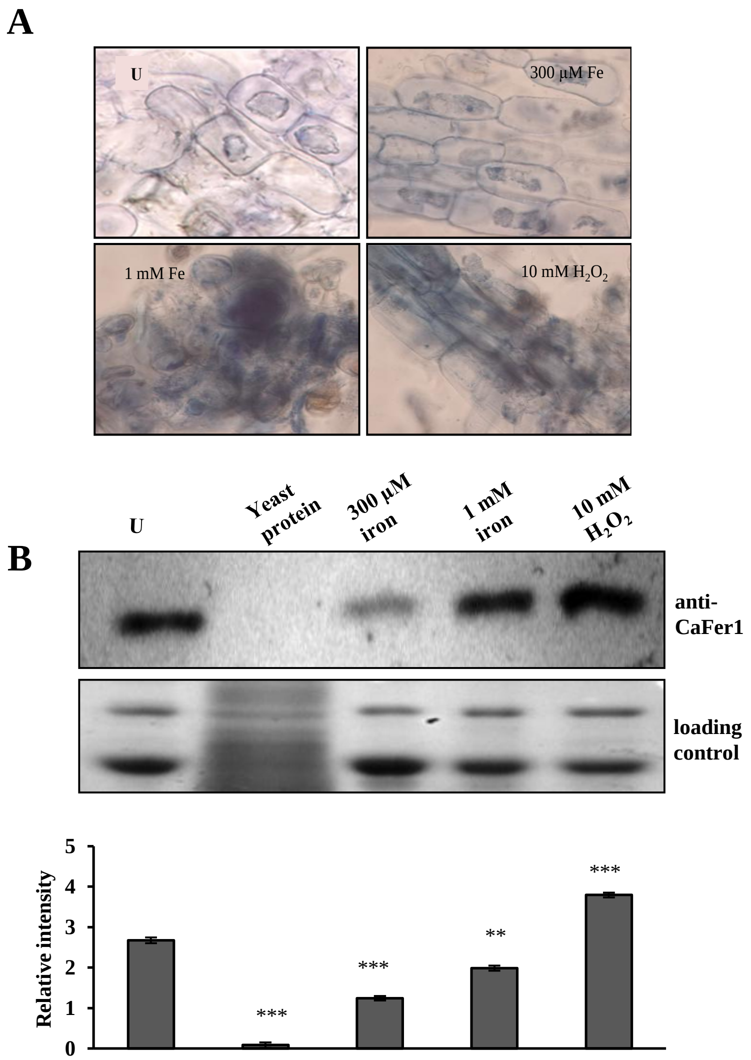

Supplementary Fig. 6. Expression of chickpea secreted ferritin influenced by iron (Fe) and H_2O_2. (A) Observation of cell death in untreated suspension culture, suspension culture treated with 300 µM Fe, 1 mM Fe and 10 mM H_2O_2. The cells were visualized under bright-field microscope. **(B)** The expression analysis of ferritin/s in the secretome in response to 300 µM and 1 mM Fe, and 10 mM H_2O_2. Yeast proteins served as the negative control. Coomassie-stained gel represents loading control. The fold-expression values are represented by bar graphs. Error bars represent the SE of three biological replicates. Asterisks indicate significant differences relative to the U background (Student's t test). U, untreated.

Supplementary Table 1: Sequences of oligonucleotide used in this study.

	Name	Sequences (5’⟶3’ FP; 3’⟶5’ RP)
qRT-PCR primers for *Arabidopsis*	AtFer1RTFP	ACTCCCTCACGGCTCTGCTT
	AtFer1RTRP	CCGCCGCCAACTTTCC
	AtFer2RTFP	TGAGGAGGTGAAGAAAGAAATGG
	AtFer2RTFRP	TGGCGAGCGAGAGAAACAA
	AtFer3RTFP	TCCATTTCGTCTCCGGTTTCT
	AtFer3RTRP	GGAAGCACGAAGAGGGAAAGA
	AtFer4RTFP	AGCTCTCTCGCTGGTGAATTTC
	AtFer4RTRP	AATGGAAGGTAACAAAGGAGACACA
	Atfh1RTFP	TCTACGGAAATTGCCGAGGTT
	Atfh1RTRP	CGCCATTGCTACGGAGAAG
	FRO2RTFP	CGATCGTTTCCTTCGGTTTC
	FRO2RTRP	AATCCGAGCAGCGAGCAA
	IRT1RTFP	GCAATCTCTCCAGCAACTTCAA
	IRT1RTRP	GTTCGCTGACTCGCTTCCA
	IRT2RTFP	GCGTTGATTACGATTGGACTGTT
	IRT2RTRP	GAGGGCCATGTAGATGAGCATT
	NRAMP1RTFP	TGCGGCTTTGGTGATTCA
	NRAMP1RTRP	AGCCAAATGTTTTCCTGTGACAA
	NRAMP 4RTFP	GGTGTGGCGACGGGTAGA
	NRAMP 4RTRP	TGCCCAAGTCGGATACTCTTCT
	UBQFP	CGACGCTTCATCTCGTCCTCC
	UBQRP	GGATCGATCTACCGCTACAACAG
	Actin4 FP	GTATGTTGCCATTCAAGCTGTTC
	Actin4 RP	GCGTAACCCTCGTAGATTGGTA
qRT-PCR primers for chickpea	CaFer1 RTFP	GCCGCTGTAACCGCTTCTT
	CaFer1 RTRP	CCAAACGCGAGTTTTCAGAATT
	CaRTEF1αFP	TCCACCACTTGGTCGTTTTG
	CaRTEF1αRP	CTTAATGACACCGACAGCAACAG
Primers of *CaFer1* for cloning in yeast vector	CaFer1FP	CACCATGCTTCTCAGAGCCGCTGTAACCGC
	CaFer1RP	TCAAGCTGCAGCTGCTTCCTCGTTGAGC
Primers of *CaFer1* for cloning in plant overexpression vectors	CaFer1FP	CACCATGCTTCTCAGAGCCGCTGTAACCGC
	CaFer1RP	AGCTGCAGCTGCTTCCTCGTTGAGCAAC

FP- Forward primer
RP-Reverse primer
RT-Real time
